# Mirk kinase inhibition targets ovarian cancer ascites

**DOI:** 10.18632/genesandcancer.19

**Published:** 2014-05

**Authors:** Xiaobing Deng, Jing Hu, Mary J. Cunningham, Eileen Friedman

**Affiliations:** ^1^ Department of Pathology, Upstate Medical University, Syracuse, N.Y., USA; ^2^ Division of Gynecologic Oncology, Department of Obstetrics and Gynecology Upstate Medical University, Syracuse, N.Y., USA

**Keywords:** Mirk/dyrk1B, kinase, ascites, ovarian cancer

## Abstract

The Mirk/dyrk1B gene is commonly amplified or upregulated in ovarian cancers, and Mirk is an active kinase in these cancers. Mirk mediates cancer cell survival by decreasing toxic ROS levels through maintaining expression of a series of antioxidant genes, possibly through its transcriptional activator functions. Mirk has the unusual property of being most active in quiescent cancer cells because of marked transcriptional downregulation by Akt/mTOR signaling and by MEK/erk signaling in cycling cells. Metastatic ovarian cancer cells form ascites, non-adherent multicellular aggregates floating within the peritoneal fluid. Most ascites cancer cells are in a reversible quiescent, dormant state, suggesting that Mirk might be expressed in these quiescent cells and thus a therapeutic target. The current studies show that ovarian cancer cell line spheroids that mimic ascites cancer spheroids were largely quiescent in G0/G1, and enriched in Mirk and the quiescence proteins, p130/Rb2 and the CDKI p27. Mirk kinase inhibition in spheroids made from established cell lines and in patient-derived ascites cancer cell spheroids reduced spheroid volume, disrupted spheroid structure to single cells, increased apoptosis, and decreased cell numbers. Earlier studies had shown that the mTOR inhibitor RAD001 increased transcription of the Mirk/dyrk1B gene, so treatments combined RAD001 with the most active Mirk kinase inhibitor. The number of ascites cells from 9 patients was reduced a similar amount by cisplatin, Mirk kinase inhibition or RAD001, but reduced substantially more, about 90%, by concurrent treatment with both the Mirk kinase inhibitor EHT5372 and RAD001. Addition of RAD001 increased the amount of toxic ROS induced by Mirk kinase inhibition. Two ascites samples taken one month apart gave similar drug responses, showing reproducibility of the techniques. Thus Mirk/dyrk1B kinase may be a therapeutic target in ovarian cancer ascites.

## INTRODUCTION

Ovarian cancer is a frequent cause of cancer death in women. Most patients with ovarian cancer have widespread disease at presentation, with yearly mortality approximately 65% of the incidence rate. The high death rate is due to widely disseminated disease at the time of diagnosis. Ovarian cancer often spreads by local shedding into the peritoneal cavity. Drug-resistant dormant ovarian cancer cells can persist for several months in the peritoneal cavity (1), (2), (3). The implantation of exfoliated ovarian cancer cells on the peritoneum leads to ascites formation and local invasion of the bowel and bladder. Tumor spheroids, non-adherent multicellular aggregates, are present in the malignant ascites of ovarian cancer patients. The majority of metastatic cells comprising spheroids within ascites fluid are quiescent, and very slow cycling. Spheroid cells exhibited minimal proliferation, with the majority [83-86%] of cells in G0/G1 by flow cytometry analysis, but spheroid cells could re-enter cell division after attachment to tissue culture dishes (4), mimicking implantation on the peritoneum. Both patient-derived and established cell line-derived spheroid cells exhibited markers of quiescence including increased levels of p130/Rb2 and the CDK inhibitor p27 (4). Thus spheroid cells are in a reversible quiescent state. Ovarian cancer initiating cells [stem cells] from primary human tumors were found within such non-adherent spheroids, exhibited enhanced chemoresistance to cisplatin or paclitaxel, and had upregulated stem cell markers such as Notch-1, Nanog, Oct-5, and cell surface markers CD117 [c-kit] (5). Thus ovarian cancer cells composing spheroids include highly malignant cells that are protected from many drugs that target dividing cells because they are in a reversible dormant state. Quiescence of tumor-initiating cells may seem counter-intuitive, but may characterize several tumor types. At any given time most leukemia-initiating cells are quiescent and therefore resistant to anticancer drugs that preferentially target dividing cells (6). Ablation of the gene Fbxw7 in leukemia-initiating cells impaired their quiescence, leading to their apoptosis (6). Identification of a druggable target protein within largely quiescent ovarian cancer ascites spheroids could have clinical utility.

Mirk/dyrk1B kinase has the unusual property of being most abundant and active as a kinase in quiescent cells and has documented activity on its characterized substrates only in quiescent cells (7),(8), (9), (10). Mutation of the Mirk gene in tumors is rare. Mirk activity increases several fold when cells leave the cell cycle and become quiescent because of poor growth conditions or following exposure to chemotherapeutic drugs like 5-FU or cisplatin (9), (11). Mirk kinase activity is also increased by K-ras or H-ras oncoproteins (12) through stress signaling to the Mirk kinase activator MKK3 (13). Mirk is expressed in about 75% of resected ovarian cancers and ovarian cancer cell lines (14),(15). Mirk/dyrk1B was found to be significantly associated with gene amplification in ovarian cancer cell lines and in high grade serous and endometrioid ovarian cancers by siRNA screening, and was critical for cell viability when amplified (16). The Mirk/dyrk1B kinase gene is localized to the 660 kb core region of the 19q13 amplicon found in a subset of pancreatic cancers and ovarian cancers (17),(18), and is amplified in various cancer cell lines, including two widely used cell lines, OVCAR3 ovarian cancer cells (14) and Panc1 pancreatic cancer cells (19). The current study was performed to determine whether Mirk/dyrk1B kinase is expressed within quiescent ovarian cancer spheroid cells from patient ascites and whether this kinase could serve as a therapeutic target.

## RESULTS

### Ovarian cancer spheroid cells are largely quiescent and express elevated levels of Mirk kinase

Mirk kinase has two main functions in cells in which it is active and highly expressed, such as many ovarian cancers. First, Mirk reduces the levels of toxic ROS by maintaining the expression of a series of at least 9 antioxidant proteins including ferroxidase and superoxide dismutase 2 (20), (11), possibly through its documented transcriptional co-activator activity (13), (21). Second, Mirk maintains some cancer cells in quiescence by phosphorylating a member of the DREAM complex that enables p130/Rb2 to sequester transcription factors (10), by stabilizing the p27 CDK inhibitor (22), and by destabilizing cyclin D isoforms (8). Mirk forms a complex with GSK3ß, and the two kinases phosphorylate cyclin D isoforms at two adjacent ubiquitination sites at T288 and T286, reducing their protein levels, and thus blocking entry into cycle. These activities enable Mirk to increase the survival capacity of dormant cancer cells. Also, transcription of the Mirk gene itself is blocked in cycling cells through Akt/mTOR signaling (23) causing Mirk to be highly expressed predominately in quiescent tumor cells. If ovarian cancer ascites cells were primarily quiescent, they might express elevated levels of Mirk and be damaged by Mirk kinase inhibition.

**Figure 1:**
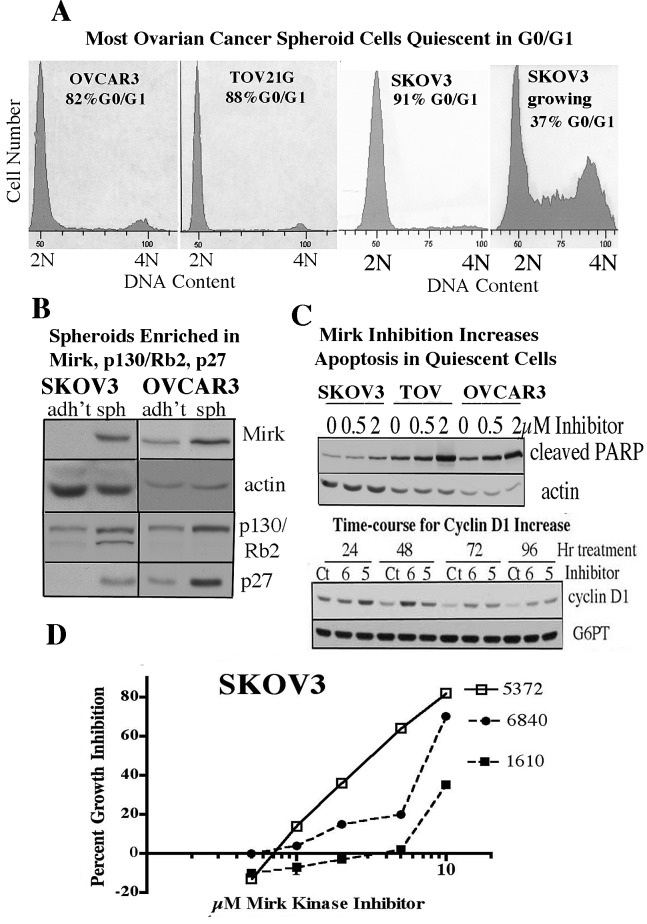
Spheroid cancer cells are quiescent and enriched in Mirk kinase A. OVCAR3, TOV21G, or SKOV3 cells were cultured according to the Spheroid Formation Protocol (Materials & Methods) in ultra-low attachment dishes in spheroid medium for 3 days to form spheroids, or allowed to grow under adherent conditions from low density for 3 days in tissue culture dishes to maintain log growth (SKOV3 growing), and analyzed by flow cytometry for DNA content, with the percent of G0+G1 cells given. B. SKOV3 and OVCAR3 cells grown under adherent (adh't) conditions or allowed to form spheroids (sph) as in panel A were analyzed by western blotting for levels of Mirk, actin, and the quiescence markers p130/Rb2 and p27. C. SKOV3 and TOV21G cells with no Mirk amplification and OVCAR3 cells with Mirk gene 20-fold amplified were made quiescent by serum-starvation to model spheroid cells, and treated with 0, 0.5 or 2µM Mirk kinase inhibitor EHT5372 for 4 days before western blotting for the apoptotic marker cleaved PARP or actin. (lower panel). Quiescent TOV21G cells were treated with two Mirk kinase inhibitors at their EC_50_ values: 6 (2.5µM EHT6840) and 5 (5µM EHT5372),for 24-96 hours and lysates examined by western blotting for cyclin D1 to measure entry into cycle and controlled by blotting for G6PT (glucose-6-phosphate translocase). D. Inverse semi-log plot showing the percentage of growth inhibition caused by Mirk kinase inhibitors compared with vehicle-treated controls, after relative cell numbers were measured by the MTT assay (n=2), mean values plotted. Adherent cultures of SKOV3 ovarian cancer cells were made quiescent by growth in serum-free DMEM while treated with a range of concentrations of the Mirk kinase inhibitors EHT5372, EHT6840 or EHT1610 for 3 days.

Spheroids were made from ovarian cancer cell lines and analyzed by flow cytometry and by biochemical analysis. Over 80% of cells comprising spheroids were in G0/G1 quiescence, while parallel cultures of growing cells had only 37% of cells in G0/G1 (Fig.1A). Spheroids made of OVCAR3 cells or SKOV3 cells were studied further as examples of tumor cells with an amplified Mirk gene (OVCAR3) or non-amplified Mirk (SKOV3) (14). Mirk protein in OVCAR3 spheroids and in SKOV3 spheroids was at 3-fold and 30-fold higher levels, respectively, than in adherent cycling cells grown in parallel cultures (Fig.1B). (14). SKOV3 and OVCAR3 spheroids had elevated levels of quiescence mediators, 2-fold (n=3) elevated levels of p130/Rb2, and 3-fold and 14-fold (n=3), respectively elevated levels of p27 compared to cycling adherent cells (Fig.1B). These data confirm studies from others showing that OVCAR3 spheroid cells were primarily in G0/G1 and exhibited increased levels of p130/Rb2 and the CDK inhibitor p27 (4). In the current study, by biochemical markers and flow cytometric analysis, most tumor cells comprising spheroids were quiescent and enriched in Mirk protein.

Recent studies using adherent cultures have shown that Mirk kinase inhibition by EHT5372 or EHT6840 reduced the number of SKOV3, OVCAR3 or TOV21G cells, all of which express active Mirk kinase (23), (24). In the current study treatment of these 3 adherent cell lines with EHT5372 led to a dose-dependent increase in apoptosis as measured by PARP cleavage, consistent with toxicity induced by high levels of ROS (Fig.1C),(data not shown). Dose-response experiments showed that the increase in apoptosis induced by each of three Mirk/dyrk1B inhibitors, EHT5372, EHT6840, and EHT1610, correlated with a decrease in the number of tumor cells (SKOV3 in Fig.1D; others not shown). Entry into cycle, as shown by an increase in cyclin D1 levels, also occurred when quiescent ovarian cancer cells were treated for 24-98 hours with either of two Mirk kinase inhibitors, EHT5372 or EHT6840 (Fig.1C, lower).

### Mirk kinase inhibition led to apoptosis of cells comprising spheroids

**Figure 2:**
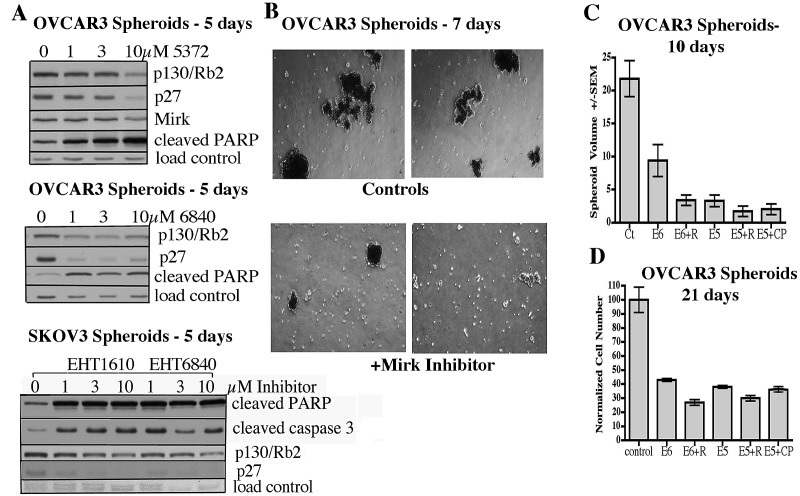
Inhibition of Mirk kinase induces apoptosis and reduces quiescence markers in two ovarian cancer cell lines made into spheroids A. OVCAR3 cells with Mirk gene amplification or SKOV3 cells with no Mirk gene amplification were cultured in ultra-low attachment dishes to form spheroids as in Fig.1A, and then treated with the Mirk kinase inhibitors EHT5372, EHT1610 or EHT6840 for 5 days. Cell lysates were examined by western blotting for levels of the quiescence markers p130/Rb2 and the CDK inhibitor p27, and the apoptosis markers cleaved PARP and cleaved caspase 3. Nonspecific bands used as the blot controls. B. Photomicrographs of representative fields of OVCAR3 spheroids after 7 days of treatment with 10µM EHT5372 using a 4x phase objective, giving 40X final magnification. Controls show clusters of spheroids. C. OVCAR3 spheroid cells were pelleted and resuspended in fresh medium containing drugs once a week, and were treated with either Mirk kinase inhibitor EHT5372 (E5) or EHT6840 (E6) at 10µM, with addition of either 5µM RAD001 or 3.3µM cisplatin, as noted. Random fields were analyzed for spheroid size after 10 days by measurement of the long and short axis of spheroids (n=10) and calculation of volumes width squared × length/2. Mean+/−SD plotted. D. Experiment in panel C continued for 21 days, and the number of OVCAR3 cells was assayed by MTT assay. Mean+/−SD shown.

If Mirk was a viable target in spheroids composed of cells from established ovarian cancer cell lines, Mirk kinase inhibition should increase ROS leading to more apoptosis and more cleaved PARP. Mirk kinase inhibition also might allow spheroid cells to leave the quiescent state, reducing their levels of p130/Rb2 and p27, so possibly sensitizing them to chemotherapeutic drugs which target cycling cells. OVCAR3 and SKOV3 spheroid cultures were treated with increasing amounts of three Mirk/dyrk1B kinase inhibitors. All three inhibitors induced up to a 5-fold increase of cleaved PARP and cleaved caspase 3 in spheroid cultures (Fig.2A), at the same time quiescence markers p27 and p130/Rb2 were decreased. Thus, inhibition of Mirk kinase activity killed some spheroid cancer cells, both in OVCAR3 cells with an amplified Mirk gene and in SKOV3 cells.

Drugs were sought that might increase spheroid cell killing in combination with Mirk kinase inhibitors. Depletion of Mirk/dyrk1B enabled low levels of cisplatin to elevate ROS levels to toxic levels in ovarian cancer cells grown under adherent conditions (11), so cisplatin was selected. The mTOR inhibitor RAD001 upregulated Mirk expression in ovarian cancer cells grown under adherent conditions and sensitized them to Mirk inhibitors (23). Possibly, RAD001 might enhance the toxicity of Mirk kinase inhibitors towards spheroid cancer cells. Thus RAD001 and cisplatin were selected along with the Mirk/dyrk1B inhibitors EHT5272 (E5) or EHT6840 (E6) for a 1- to 3-week treatment of OVCAR3 spheroids. After 1 week, spheroids remained in all cultures, but more single cells were seen in the Mirk kinase inhibitor treated cultures and some of the spheroids looked smaller (Fig.2B), as expected from the induction of apoptosis markers. After 10 days of culture, 10 randomly selected spheroids in each culture were measured by microscopy using an eyepiece micrometer. The average volume of the OVCAR3 spheroids in cultures treated with EHT6840 plus RAD001, EHT5372 alone or with RAD001 or cisplatin was 7-fold smaller than the untreated control cultures (Fig.2C), indicating that the treatments strongly decreased spheroid cell growth and led to spheroid dissociation. After 3 weeks of growth, with fresh media and drugs added weekly, cell numbers in the spheroids were measured (Fig.2D). Because of limited penetration of the dye, only the outer spheroid cells were counted, decreasing the sensitivity of the assay, and in particular, leading to an overestimation of the number of control cells. However, the spheroids treated with EHT6840 or EHT5372 had at most half as many cells as the controls, while addition of RAD001 further reduced measurable cell numbers. Addition of cisplatin to the Mirk kinase inhibitors had minimal effects. Visualization of the 3 week cultures by microscopy showed that the size of the spheroids was smaller in the cultures treated with either Mirk kinase inhibitor, EHT6840 or EHT5372 (data not shown). Thus two Mirk/dyrk1B kinase inhibitors reduced the size of OVCAR3 spheroids, with a modest increase in efficacy by addition of the mTOR inhibitor RAD001.

### Time-course shows Mirk kinase inhibition over a 3-week period can lead to massive apoptosis of patient-derived ovarian cancer ascites spheroids

**Fig.3:**
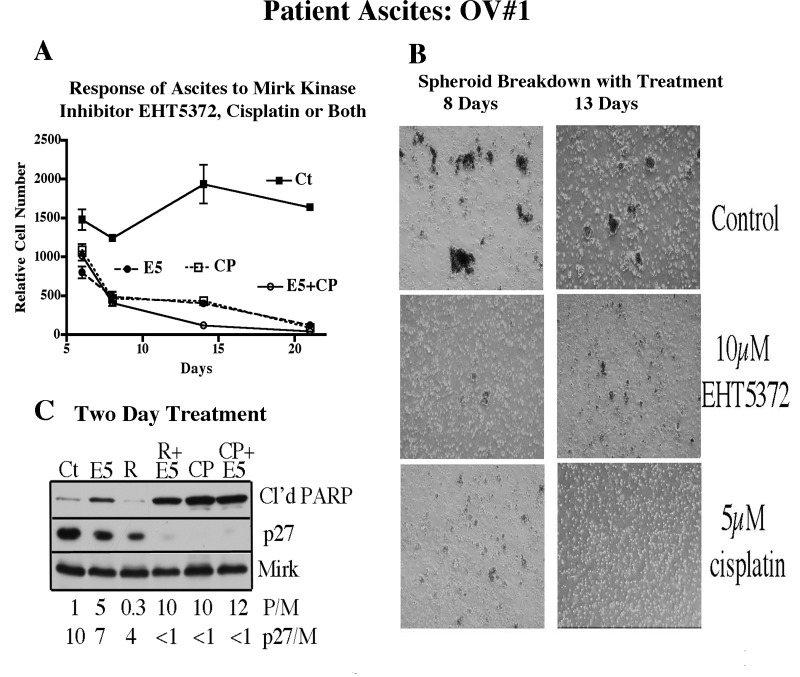
Mirk kinase inhibitor reduces ascites spheroids of ovarian cancer patient #1 to single cells, which then undergo apoptosis Ascites were processed (Methods & Materials), and equal aliquots were placed in 6-well low attachment tissue culture wells. After 24 hours, the ascites were treated with either 10µM Mirk/dyrk1B kinase inhibitor EHT5372, 5µM RAD001, 5µM cisplatin, or combinations as noted. A. Ascites spheroids were pelleted and resuspended in fresh medium containing drugs once a week. After 6, 8, 14 and 21 days, the number of cells was assayed. Mean+/−SD shown. B. Photomicrographs of ascites spheroids after 8 and 13 days of treatment. C. Lysates were examined by western blotting after the ascites were treated for 2 days. The ratios of cleaved PARP to Mirk or p27 to Mirk are given below the respective lanes.

Cancer cells alter gene expression to adjust to tissue culture conditions, which may modify sensitivity to putative therapeutic agents. Culture of cancer cells in adherent conditions in tissue culture plates also alters gene expression. For these reasons, we studied ascites cancer spheroids taken directly from patients in the operating room and maintained the cancer cell spheroids in suspension culture. Ascites OV#1 cancer spheroid cell cultures remained viable in culture for at least 21 days and total cell number did not decrease over this time period, as shown by metabolism of MTT after 6,8,15, and 21 days, with 3 separate analyses per point (Fig.3A). The MTT assay was chosen because a retrospective analysis of 120 epithelial ovarian cancer patients showed that the MTT assay results of chemosensitivity of *in vitro* ascites assays were consistent with the clinical response and the time to progression (25). The ascites were treated with the Mirk kinase inhibitor EHT5372 at 10 µM, the concentration optimal for inducing apoptosis (Fig. 2). It was also important to select a concentration that was effective in vivo against tumor cells, without killing the host. At 10µM, EHT5372 injected intraperitoneally into athymic mice reduced the size of Panc1 xenografts without causing any weight loss in the mice and the mice appeared normal and lively (Deng and Friedman, manuscript in preparation).

Ascites were also treated with the mTOR inhibitor RAD001 at 5µM, a concentration used in vivo, and as a positive control, cisplatin at 5µM, a level much higher than that used clinically. The association of ascites cells into loose multicellular “spheroids” was maintained in the nonadherent culture conditions used, while spheroids treated with the Mirk kinase inhibitor or cisplatin were largely reduced to single cells (Fig.3B). Interestingly, others have reported that stable short hairpin RNAs against Mirk(dyrk1B) prevented the A598 renal carcinoma cell line from forming spheroid cultures (26), consistent with our observations of the loss of spheroid structure with Mirk kinase inhibition. The Mirk/dyrk1B inhibitor EHT5372 caused a progressive loss in tumor cell numbers: 50% after 6 days, 60% after 8 days, 80% after 15 days, and 92% after 21 days (Fig.3A). The Mirk kinase inhibitor alone was as effective as the high dose of cisplatin.

The effect of a 2-day treatment with these agents was measured by western blotting for the apoptosis marker cleaved PARP (Fig.3C). The Mirk kinase inhibitor induced a 5-fold increase in cleaved PARP, which was doubled by addition of RAD001. Most ovarian ascites cells are in a quiescent G0/G1 state characterized by high levels of the CDK inhibitor p27 (4). The Mirk/dyrk1B kinase inhibitor, RAD001, and cisplatin individually reduced p27 levels, which were made undetectable by the drug combinations, suggesting that ascites cells entered cycle.

### Summary of Patient Cases

**Fig.4:**
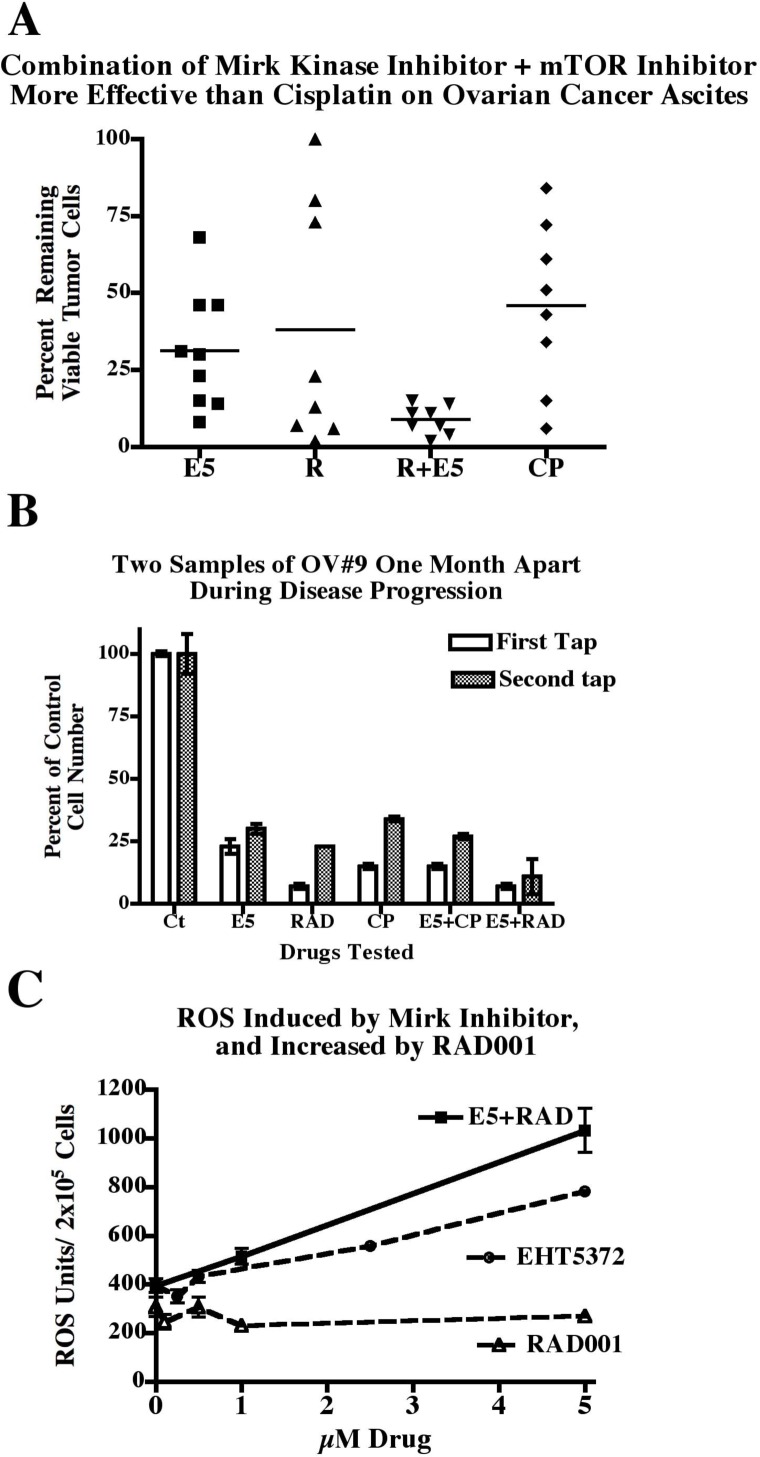
Summary of the effects of Mirk kinase inhibitor and mTOR inhibitor on ovarian ascites cell numbers from patients, and increase in ROS induction by Mirk kinase inhibitor when co-treated with RAD001 A. Summary of the percent of remaining viable ascites tumor cells from 9 patients after treatment with 10µM EHT5372, 5µM RAD001, the combination, or 5µM cisplatin. Mean value bars shown. B. Ascites samples from patient #9 were removed two times, one month apart, with the later one shown on the right bars. Both were tested for 28 days with drugs as above before analysis by uptake of MTT. Mean+/−SD shown if SD>5%. C. One day post-plating, parallel sets of TOV21G ovarian cancer cells were treated for 1 day with increasing concentrations of either the Mirk kinase inhibitor EHT5372 or the mTOR inhibitor RAD001, or both compounds, in DMEM containing 0.2% FBS. Half of the cell sets were then incubated 30 min with the ROS indicator 5µM CM-DCFDA in phenol-red free, serum-free DMEM, and all sets of cells were incubated in DMEM+5%FBS for 30 min, then trypsinized and fluorescence and cell number measured. Mean+/−SD shown.

To quantitate the effects of the various drugs, the percentage of ascites cells remaining after treatment in each case was plotted (Fig.4A). For the nine patient samples in which drug effects were evaluated by cell numbers after the longest drug exposure, usually 3 weeks, a mean of 31%+/−6% (SE) of tumor cells remained after treatment with the Mirk kinase inhibitor EHT5372, a mean of 46%+/−10% remained after treatment with the elevated dosage of cisplatin, a mean of 38%+/−14% remained after treatment with RAD001, and a mean of 9%+/−2% of tumor cells remained after concurrent treatment with the Mirk kinase inhibitor and the mTOR inhibitor RAD001(Fig. 4A). Thus the kinase Mirk/dyrk1B appears to be a new therapeutic target in ovarian cancer ascites, in particular when added together with the mTOR specific inhibitor RAD001 (everolimus), a drug already in clinical use (27), (28).

Ascites are drained from patients to relieve discomfort. Ascites were removed from one patient two times, at a one month interval, and both samples were treated for 4 weeks in vitro. Significantly, the general pattern was similar, suggesting that our method gave reproducible results. The second ascites sample was more drug resistant as expected from patient studies, with the percentage of cells cisplatin resistant increasing from 15% to 27% (Fig.4B). However, the combination of the Mirk kinase inhibitor and the mTOR inhibitor RAD001 killed most cells at both time points.

Was the increase in ascites cell death when the mTOR inhibitor RAD001 was added to the Mirk kinase inhibitor (Fig.4A) due to an increase in toxic ROS levels? A series of mTOR inhibitors increased Mirk expression several fold in ovarian cancer cells (23), and Mirk depletion or kinase inhibition increases ROS levels (20), (11). TOV21G cells were treated with increasing concentrations of the Mirk kinase inhibitor EHT5372, the mTOR inhibitor RAD001, or combinations of both compounds at 1µM or 5µM. EHT5372 increased ROS levels in a dose-dependent manner, while RAD001 did not, with the amount of ROS in the RAD001 treated cells identical to that in untreated cells (Fig.4C). The combination of 5µM EHT5372 and 5µM RAD001 generated more ROS than EHT5372 alone, 1000 units to about 700 units. Thus, the increased toxicity of ovarian ascites cancer cells to the combined Mirk kinase inhibitor and mTOR inhibitor could be attributed to the increase in ROS.

### Were ascites cancer cells killed by the Mirk kinase inhibitor because Mirk was the major target?

No kinase inhibitor is completely specific so it is possible that off-target effects were responsible for the ascites cancer cell killing by the Mirk kinase inhibitor. Depletion of Mirk mRNA within ascites spheroids was not attempted because of technical difficulties in reaching all of the cells comprising the tumor spheroids. In contrast, a small molecule kinase inhibitor capable of diffusing into cells is expected to reach most spheroid cells. Earlier studies had already shown that Mirk depletion in each of four ovarian cancer cell lines grown adherent increased their toxic ROS levels by reducing expression of several antioxidant genes including superoxide dismutase 2 and ferroxidase (11), results consistent with the current studies in which spheroid cells made from two of these lines, OVCAR3 and SKOV3, were killed by Mirk kinase inhibition. In addition, ROS levels increased when Mirk kinase was inhibited and further increased when ovarian cancer cells were also treated with RAD001 (Fig.4C), showing similar effects of both Mirk depletion (11) and Mirk kinase inhibition by EHT5372.

**Fig.5:**
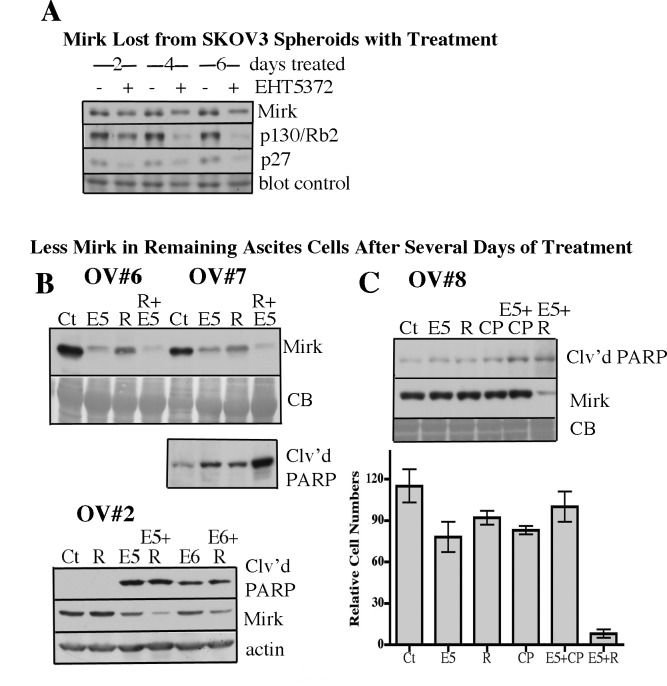
Loss of Mirk expression from ascites spheroid cells correlates with increased apoptosis A. Western blot of Mirk, p130/Rb2 and p27 in SKOV3 spheroids treated for 2-6 days with 10µM EHT5372. B. Western blot of Mirk in remaining cells of OV#6 and OV#7 after 7 day treatment of ascites, and OV#2 after a 6 day treatment, with either 10µM Mirk kinase inhibitor EHT5372 (E5), 10µM EHT6840 (E6) 5µM mTOR inhibitor RAD001, or combinations, as noted. CB, coomassie blue stained blot as blot control. Separate western blot of OV#7 of PARP cleavage. C. Western blot analysis of cleaved PARP and Mirk in remaining cells of OV#8 after 5 days of treatment. 5µM cisplatin added as noted. CB, coomassie blue stained blot as blot control. (lower panel) Relative OV#8 cell number (mean+/−SD) by MTT analysis after 6 days of treatment.

Another approach was taken to determine whether Mirk-expressing cells were targeted in patient cancer ascites. Mirk protein levels vary up to 10-fold throughout the cell cycle, with the highest levels in quiescent cells, as Mirk expression is blocked by Akt/mTOR signaling (23) and MEK/erk signaling (29). Therefore, cancer cells within the same tumor express different amounts of Mirk protein. Possibly ascites cancer cells expressing the most Mirk would be preferentially lost from the cultures after Mirk kinase inhibition since OVCAR3 spheroid cells treated for 5 days with 10µM EHT5372 expressed less Mirk protein and more cleaved PARP than control cells (Fig.2A, last lane). Also, Mirk inhibitor treatment reduced Mirk levels about 2 fold in SKOV3 spheroids (Fig.5A).

Initially, we observed that a 2-day treatment of OV#1 ascites with the Mirk inhibitor with or without RAD001 led to only a 20-25% decrease in Mirk protein levels (Fig.3C). However, a longer treatment reduced Mirk levels in the remaining ascites. Four ascites, OV#2, OV#6, OV#7, and OV#8 were analyzed for Mirk expression 5 to 7 days after initiation of ascites treatment (Fig.5B&C). The OV#6 and OV#7 ascites cells remaining after Mirk kinase inhibitor EHT5372 treatment exhibited 6-9% as much Mirk protein as controls, and the OV#2 ascites cells had 50% as much. Loss of Mirk protein correlated with an increase in cleaved PARP in OV#7 and OV#2 ascites cells treated with Mirk kinase inhibitor with or without the addition of RAD001. The combination of EHT5372 plus RAD001 left cells with 1-15% as much Mirk protein as controls, and a 5-fold increase in PARP cleavage (Fig.5B). In the OV#8 ascites, Mirk levels were not reduced in the tumor cell spheroids treated with only the Mirk kinase inhibitor, but little apoptosis and little cell loss occurred in this treatment (Fig.5C). Reduction in Mirk protein, reduction in cell number and an increase in cleaved PARP were seen in ascites OV#8 treated with both the Mirk inhibitior and RAD001. Thus, treatment of ascites cancer cell spheroids with the Mirk kinase inhibitor with or without RAD001 left tumor cells expressing little Mirk protein only if apoptosis and/or loss of spheroid cells was seen. These correlations suggest either that the Mirk kinase inhibitor targeted ascites cancer cells with the highest Mirk levels, or that the Mirk kinase inhibitor forced movement of the quiescent ascites cells into cycle with a concomitant decrease in Mirk expression.

### Movement into cycle from the quiescent state was not necessary for Mirk kinase inhibition to kill ovarian cancer ascites cells

**Fig.6:**
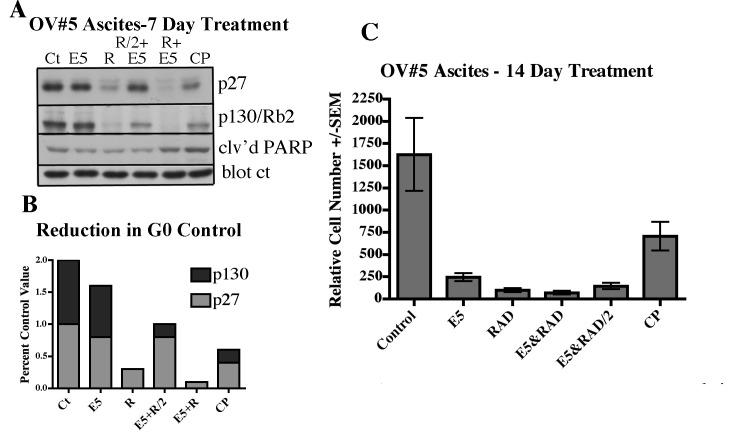
Effect on G0 proteins p27 and p130/Rb2 by treatment of OVAS#5 with Mirk kinase inhibitor A. OVAS#5 treayed for 7 days with Mirk kinase inhibitor EHT 5372, RAD001 or cisplatin, each at 10µM, except for E5+R2/2 which was E5 and RAD001 at 5µM each. Lysates were examined by western blotting. B. The ratios of p27 or p130/Rb2 to a cross-reacting band used as the control are shown graphically below the respective lanes after densitometry analysis. C. Relative cell number by MTT analysis after 14 days.

OV#5 was treated for 7 and 14 days with the Mirk kinase inhibitor EHT5372, and the same other drugs as used previously (Fig.6). EHT5372 had little effect on levels of the quiescence proteins p130/Rb2 and p27 after 7 days (Fig.6A), but reduced cancer cell numbers 8-fold after 14 days (Fig.6B), suggesting that it was not necessary for a Mirk kinase inhibitor to release tumor cells from quiescence to kill them.

## DISCUSSION

Quiescent cancer cells are difficult to target with conventional chemotherapy and radiation therapies, which preferentially kill cycling cells. Moreover, drug-resistant dormant ovarian cancer cells persist for several months in the peritoneal cavity (1), (2), (3). Other investigators have shown that most metastatic ovarian ascites cancer cells are in a dormant, reversibly quiescent state (4), and such cells can go back into cycle when they implant on the peritoneum. Mirk kinase is either amplified or upregulated in the majority of ovarian cancers, and has the unusual property of being most abundant in quiescent cells, so might be expressed in ovarian cancer ascites. The current study has shown Mirk is enriched in spheroids made from ovarian cancer cell lines, these spheroids are composed of quiescent cells, and that each of three Mirk kinase inhibitors can kill these quiescent spheroid cells. Mirk maintains the survival of quiescent cancer cells, ovarian, pancreatic or colon (14), (20). For example, stable overexpression of Mirk enabled colon cancer cells, made quiescent by growth factor deprivation, to survive better than parallel cultures stably overexpressing kinase-dead Mirk (29). The cell line spheroids served as models for patient ascites cancer.

The current study also demonstrated that Mirk kinase could be targeted in ovarian cancer ascites from patients by a small molecule inhibitor. Ascites from 11 patients were analyzed, with cell number assayed by MTT metabolism in 9 cases. In six of the 11 cases, enough material was received to allow western blotting and in each case Mirk protein was detected and growth inhibition by a Mirk kinase inhibitor was also seen, suggesting at least some specificity. The Mirk kinase inhibitor caused some cell kill in each of these 11 metastatic cancers, averaging about 70% kill, with a wide distribution, better than the mean 54% cell kill caused by cisplatin. Cell kill was measured by metabolism of MTT, which would underestimate the number of untreated control cells because of poor penetration of dye beyond the outer layer of cells, so cell kill is probably larger. Earlier studies showed that inhibitors of mTOR increase Mirk kinase expression, and the combination of a Mirk kinase inhibitor and the mTOR inhibitor RAD001 killed more SKOV3 and TOV21G ovarian cancer cells than either agent alone (23). In the current study RAD001 and the Mirk kinase inhibitor EHT5372 were more effective together than either agent alone against ovarian cancer ascites cells, killing more than 90%. The PI3K/Akt/mTOR signaling pathway is one of the most frequently deregulated signaling pathways in solid tumors including ovarian cancers, as compiled in the Cancer Genome Atlas, and has a functional role in drug resistance. RAD001 [everolimus] also has an anti-angiogenic effect in an ovarian cancer xenograft model (30), and RAD001 was shown to delay tumor onset and progression in a transgenic mouse model of ovarian cancer (31). Targeting mTOR may overcome cisplatin resistance in ovarian cancer patients and an early response to everolimus treatment was seen in an animal model of cisplatin-resistant ovarian cancer (32). Interestingly, ROS can activate the mTOR pathway (33). RAD001 is used clinically against a small selection of tumors, but mTOR inhibitors activate tumor survival pathways by complex feedback mechanisms. Since Mirk kinase mediates survival of some cancers, its inhibition might increase the utility of mTOR inhibitors in these cancers.

Possibly the 10µM concentration of Mirk kinase inhibitor EHT5372 that induced the death of ascites cancer cells in vitro would be toxic to normal cells in vivo. This was not the case because at the same time the current study was being performed, ten 8-week old athymic mice, mean size 30 g, were given intraperitoneal injections of EHT5372 to give a 10µM final concentration, or injected with the PBS dilution. The injections were given twice weekly over a 2 week period. The weight of the control mice averaged 29+/1 g, identical to the average weight of the Mirk kinase inhibitor treated mice, 30.8+/−1.6g and the mice appeared normal and remained lively. The mice were bearing xenografts of Panc1 pancreatic cancer cells, and the size of the xenografts was decreased, showing that the Mirk kinase inhibitor was active in vivo against cancer cells (Deng and Friedman, manuscript in preparation). Thus the Mirk kinase inhibitor concentration that strongly inhibited the viability of ovarian ascites spheroids in suspension culture within 2-4 weeks and induced their apoptosis also decreased the size of Panc1 xenografts in vivo without detectable toxicity in the mice.

This lack of toxicity of the Mirk kinase inhibitor in mice was not a surprise. Embryonic knockout of the Mirk/dyrk1B gene is not lethal (34). The Mirk kinase inhibitor RO5454948 in earlier studies had no detectable toxicity on cultured normal diploid fibroblasts or normal diploid ovarian epithelium from human donors (24). At identical concentrations and exposure times the Mirk kinase inhibitor RO5454948 was toxic to ovarian cancer cells with low or absent expression of the CDK inhibitors p16 and p21, the latter because of mutated, inactivated p53, a mutation seen in almost all ovarian cancers. Mirk's mechanism of action helps to explain these disparate effects. Mirk/dyrk1B mediates the stability of the DREAM complex that holds cells in G0 (10). Mirk kinase also helps to maintain cells in quiescence by stabilizing p27 and by destabilizing cyclin D isoforms. As a result, Mirk kinase inhibition stabilizes cyclin D1 and cyclin D3 in ovarian cancer cells (24), as well as in several other types of cells. The elevation in cyclin D levels in ovarian cancer cells with low levels of G1 CDK inhibitors unbalances G1 cell cycle regulation and enables a premature exit from the quiescent state, with a loss of sequestered E2F4 from p130/Rb2, with subsequent apoptosis and cell death (24). Premature entry into cycle after Mirk kinase inhibition or depletion led to an increase in toxic ROS species in earlier studies, while treatment with an ROS scavenger reduced the amount of cleaved PARP and the extent of cancer cell loss. Normal diploid cells with the usual complement of CDK inhibitors were able to resist such a premature entry into cycle after Mirk kinase inhibition (24). Mirk/dyrk1B has low expression and low kinase activity in most normal tissues including normal diploid ovarian epithelial cells (29), (24). Thus pharmacological inhibition of Mirk/dyrk1B should predominantly affect cancer tissues.

## MATERIALS AND METHODS

Cell lines, media, western blotting, MTT assays, flow cytometry as described (14). Cell lines and strains were obtained from the ATCC, and fresh cells were taken from frozen stocks negative for mycoplasma, on average every 3 months. STR (short tandem repeat) profiling of 14 and 15 loci, respectively, authenticated the SKOV3 and TOV21G cell lines.

### Mirk/dyrk1B inhibitors:

EHT5372, EHT1610, and EHT6840 were gifts from Diaxonhit, and were patented: Leblond B., Casagrande A.-S., Désiré L., Foucourt A., Besson T., DYRK1 inhibitors and uses thereof, WO 2013026806, 2013.02.28. RAD001 and cisplatin were purchased from Selleck Chemical. All other reagents were from Sigma. EHT5372 was profiled at 250nM against a panel of 339 kinases including all of the dyrk family members and was highly selective (35).

### Spheroid culture preparation from cell lines:

1 million cells are plated in a 100mm tissue culture dish in 10ml spheroid media and allowed to attach and grow for at least 2 days. Cells are then trypsinized, washed twice, then suspended in 10 ml spheroid media (DMEM/F12 supplemented with 0.5% BSA, 5 µg/ml insulin, 10 ng/ml basic FGF and 20 ng/mg EGF) and placed in an ultra-low attachment dish (Fisher), and in 2-3 days spheroid formation occurred. Viable cell numbers in spheroids and patient ascites were determined by the MTT metabolism.

### Ovarian cancer patient ascites:

Specimens were obtained from patients with newly diagnosed epithelial ovarian cancer.1 liter of ascites was centrifuged in conical tubes to pellet the malignant cells. The red blood cell layer was aspirated and the remaining tumor cells were resuspended in fresh ovarian cancer spheroid medium, and directly aliquoted into multiple 6-well low attachment tissue culture dishes. The malignant cells remained associated as loose multicellular “spheroids”. Patient samples were obtained after patient consent under a protocol approved by the Institutional Review Board.
